# Genetic mutations in non-syndromic deafness patients of uyghur and han chinese ethnicities in xinjiang, China: a comparative study

**DOI:** 10.1186/1479-5876-9-154

**Published:** 2011-09-14

**Authors:** Yu Chen, Mayila Tudi, Jie Sun, Chao He, Hong-li Lu, Qing Shang, Di Jiang, Pilidong Kuyaxi, Bin Hu, Hua Zhang

**Affiliations:** 1 Department of Otorhinolaryngology, the First Affiliated Hospital of Xinjiang Medical University, Urumqi 830054, PR China; 2 Department of Otorhinolaryngology, the First People's Hospital, Kashi Municipality (Xinjiang), PR China; 3 Department of Laboratory Medicine, the First Affiliated Hospital of Xinjiang Medical University, Urumqi, PR China; 4 National Engineering Research Center for Biochip Technology, Beijing, PR China

## Abstract

**Background:**

The deafness-associated gene mutation profile varies greatly among regions and races. Due to the multi-ethnic coalition of over one thousand years, non-syndromic deafness (NSD) patients of Uyghur ethnicity may exhibit a unique deafness-associated gene mutation spectrum as compared to Han Chinese deaf population.

**Methods:**

In order to characterize nine loci of four deafness-associated genes of Uyghur NSD patients in comparison with Chinese Han deaf population, NSD patients (n = 350) were enrolled, including Uyghur (n = 199) and Han Chinese (n = 151). Following the history taking, blood samples were collected for DNA extraction. DNA microarray was performed on nine loci of four deafness-associated genes, including 35delG, 176-191del16, 235delC, 299-300delAT, 538C > T, 1555A > G, 1494C > T, 2168A > G, and IVS7-2A > G. The samples that showed the absence of both wild and mutant probe signals were tested for further DNA sequencing analysis.

**Results:**

The mutations in the nine loci of prevalent deafness-associated genes were detected in 13.06% of Uyghur NSD patients and 32.45% of Han Chinese patients (*P *< 0.05), respectively. *GJB2 *mutation was detected in 9.05% of Uyghur patients and 16.56% of Han Chinese patients (*P *> 0.05), respectively. 235delC was the hotspot mutation region in NSD patients of the two ethnicities, whereas 35delG was the mutation hotspot in Uyghur patients. 187delG mutation was detected for the first time in Uyghur NSD patients and considered as an unreported pathological variant of *GJB2. SLC26A4 *mutation was found in 2.01% of Uyghur patients and 14.57% of Han Chinese patients (*P *< 0.05), respectively. The frequencies of mtDNA 12S rRNA mutation in Uyghur and Han Chinese patients were 2.01% and 2.65% (*P *> 0.05), respectively. The NSD patients exhibited a low frequency of *GJB3 *mutation regardless of ethnicity.

**Conclusion:**

Prevalent deafness-associated gene mutations in the nine loci studied were less frequently detected in Uyghur NSD patients than in Han Chinese patients. *GJB2 *was the most common mutant gene in the two ethnicities, whilst the two ethnicities differed substantially in hotspot mutations. A low-frequency *SLC26A4 *mutation was detected in Uyghur NSD patients. Uyghur NSD patients differed significantly from Han Chinese patients in gene mutation profile.

## Background

Deafness is a worldwide prevalent disease that seriously impairs human quality of life. The global incidence of congenital deafness is 1-3 per 1,000 newborns. The epidemiological studies show that one half of deafness at childhood is associated with genetic factors [1,2]. Non-syndromic deafness (NSD) is the most common neurosensory deafness, and accounts for 70% of inherited hearing impairment [3]. Until now, more than 200 genes have been associated with NSD, including 36 autosomal recessive genes, 24 autosomal dominant genes, and two X-linked genes (http://hereditaryhearingloss.org/, updated in October 2010).

The deafness-associated gene mutation spectrum as well as dominant gene profile vary greatly among regions and races [4,5]. 35de1G, 167de1T, and 235de1C are reported to be the most prevalent mutant genes among Caucasian, Jewish, and Asian populations, respectively [6-8]. The epidemiological studies demonstrate that a large proportion of NSD can be caused by a relative small number of mutant genes, including *GJB2, SLC26A4*, and mtDNA 12S rRNA [9-13]. A positive result is very likely to be obtained by scanning the hotspot mutations of these genes alone. Microarray chip technology is a high-throughput and high-efficiency tool for the detection of gene mutations. CapitalBio Corporation (Beijing, China) develops a microarray chip that is designated to detect mutation hotspots of inherited deafness in Chinese population, based on the large-scale epidemiological study data across 28 provinces and municipalities from Chinese People's Liberation Army General Hospital [14-16]. This microarray chip allows the simultaneous detection of nine loci of four deafness-associated genes, namely, 35delG, 176-191del16, 235delC and 299-300delAT for *GJB2 *gene, 538C > T for *GJB3 *gene, 1555A > G and 1494C > T for mtDNA 12S rRNA gene, and 2168A > G (H723R) and IVS7-2A > G for *SLC26A4 *gene.

Xinjiang is located on the northwest border of China, where central and western Asian cultures converge. It is a region populated by a variety of ethnicities but mainly by Uyghur ethnicity. The deafness-associated gene mutation spectrum and dominant mutation profile may be unique in Uyghur population residing in Xinjiang due to the multi-ethnic coalition over one thousand years. In this study, we use the aforementioned microarray chip to examine and analyze nine loci of four deafness-associated genes among Uyghur and Han Chinese NSD patients residing in Xinjiang, in aiming to characterize nine loci of four deafness-associated genes of Uyghur NSD patients in comparison with Han Chinese patients.

## Materials and methods

### Patients and DNA samples

The study protocol was approved by the Institutional Review Board at the First Affiliated Hospital of Xinjiang Medical University. The subjects, or their legal guardians in the case of subjects under 18 years, volunteered to give informed consent. Subjects were enrolled from the representative deaf populations registered with Xinjiang local institutes, including Kashi Municipal Special Education School, Kashi Municipal Disabled People's Association, Changji Municipal Special Education School, Urumqi Lingual Training School, and Otorhinolaryngology Outpatient Clinic, the First Affiliated Hospital of Xinjiang Medical University. Parents were inquired for a full history, including family history, pregnancy/labor process, age of onset/diagnosis, and previous history of infection, head trauma and medication with aminoglycosides. Deaf patients received routine physical and otorhinolaryngoloical examination, as well as pure tone audiometry. Brainstem auditory evoked potential test was performed in uncooperative children. Only biologically unrelated NSD patients (n = 350) who exhibited bilateral moderate or severe sensorineural hearing impairment on pure tone audiometry or brainstem auditory evoked potential test were included. Parents of NSD children were excluded from this study. The ethnicity was determined based on the institutional household records. Healthy volunteers of both Uyghur (n = 103) and Han Chinese ethnicities (n = 70) who resided in Xinjiang and exhibited normal hearing were enrolled as control subjects. Peripheral venous blood samples (3-5 ml) were collected from both NSD patients and control subjects.

### Mutational analysis with PCR

Genomic DNA was extracted from peripheral blood leukocytes using a commercial available DNA isolation kit (Tiangen Biotech Corporation, Beijing, China). The concentration and purity of DNA were determined using an ultraviolet spectrometry (ActGene Inc, Taipei, Taiwan). Polymerase chained reaction, chip hybridization, and chip scan were conducted as recommended by the manufacturer of the microarray kit (CapitalBio Corporation, Beijing, China) for the simultaneous detection of nine hotspot mutations in four most prevalent NSD-associated genes, including *GJB2 *(35delG, 176-191del16, 235delC, and 299-300delAT), *GJB3 *(538C > T), *SLC26A4 *(IVS7-2A > G and 2168A > G), and mitochondrial 12S rRNA (1555A > G and 1494C > T). The test results were determined based on the fluorescent hybridization signal and the distribution of microarray probe. In the case of the deletion at any locus, the DNA sample was further sequenced by using an ABI 3730XL analyzer (ABI, Foster, USA).

### Statistical analysis

The statistical analysis was performed using SPSS 17.0 (SPSS Inc., Chicago, USA). The intergroup difference in frequency was compared using the two-tailed chi-square test or Fisher's exact probability test. A *P*-value less than 0.05 was considered statistically significant.

## Results

In this study, the NSD population (n = 350) included 199 Uyghur patients (86 males and 113 females), aged 12.7 ± 5.3 years (range, 1-35), and 151 Han Chinese patients (86 males and 65 females), aged 10.9 ± 5.4 years (1-26). Out of 350 patients, 33 patients had a family history of deafness, whose siblings, parents, or other close relatives were also deaf, including 22 Uyghur and 11 Han Chinese subjects. The remaining 317 cases were determined to be sporadic.

### Prevalent deafness-associated gene mutations in NSD patients and control subjects

**Figure 1:**
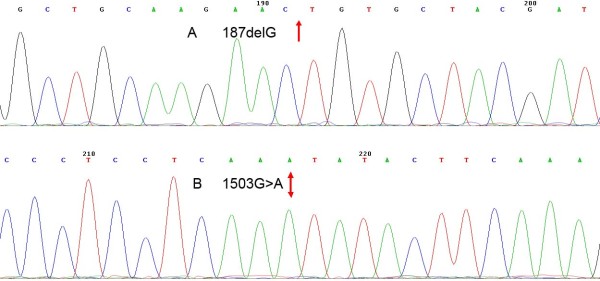
**DNA sequence chromatograms of homozygous mutations in patients with nonsyndromic deafness: (A) 187delG and (B) 1503G > A**.

Out of 199 Uyghur patients, 22 (11.1%) patients exhibited mutations in one allele or more. The deletion of both mutant and wild probe signal was detected in four (2.0%) Uyghur patients. This suggested that the possible presence of new mutant locus failed the effective binding of hybridization prime to the DNA sequence of the test sample. The further gene sequencing identified 187delG/187delG for *GJB2 *in three patients (Figure 1A), and 1503G > A for mtDNA 12S rRNA in one patient (Figure 1B). The homogenous mutations identified in these four patients were, therefore, included as positive test results (Table 1). The frequencies of prevalent deafness-associated gene mutations in the nine loci were 13.06%, 1.94%, 32.45% and 4.28% in Uyghur patients, Uyghur control subjects, Han Chinese patients, and Han Chinese control subjects, respectively (Table 2). The mutation frequency was significantly lower in Uyghur patients than in Han Chinese patients (χ^2 ^= 19.162, *P *< 0.05). Moreover, the mutations were also more frequently detected in the patient population than in control subjects for either ethnicity (χ^2 ^= 9.983, *P *< 0.05; χ^2 ^= 21.086, *P *< 0.05).

**Table 1:** Genotypes of deleted loci in patients with nonsyndromic deafness

Deleted loci	Mutant loci confirmed on sequencing analysis	Samples size
176-191del16 (*GJB2*)	187delG/187delG	3
1494C > T (12S rRNA)	1503G > A	1

**Table 2:** Mutation frequencies of prevalent genes in Uyghur and Han Chinese patients with nonsyndromic deafness and control subjects

Study subjects	n	n of mutation	Rate
Uyghur patients	199	26	13.06%
Han Chinese patients	151	49	32.45%
Uyghur control subjects	103	2	1.94%
Han Chinese control subjects	70	3	4.28%

### Multination of deafness-associated gene loci in NSD patients and control subjects

The frequencies of mutations for *GJB2, SLC26A4*, mtDNA 12S rRNA and *GJB3 *in the two ethnicities were shown in Table 3. The mutant genotypes of the four deafness-associated genes in Uyghur patients, Uyghur control subjects, Han Chinese patients, and Han Chinese control subjects were presented in Table 4.

**Table 3:** Detection rates of deafness-associated gene mutations among Uyghur and Han Chinese patients with nonsyndromic deafness

Patient group	n	Number of positive samples (%)			
		
		*GJB2*	*SLC26A4*	12S rRNA	*GJB3*
Uyghur	199	18 (9.05%)	4 (2.01%)	4 (2.01%)	0 (0.0%)
Han Chinese	151	25 (16.56%)	22 (14.57%)	4 (2.65%)	2 (1.32%)

**Table 4:** Genotypes of prevalent gene mutations in nonsyndromic deafness patients and control subjects of Uyghur and Han Chinese ethnicities

Genotype	Uyghur patients (n)	Han Chinese patients (n)	Uyghur control subjects (n)	Han Chinese control subjects (n)
235delC/-	4	9	1	2
235delC/235delC	3	6	0	0
35delG/-	3	0	0	0
35delG/35delG	1	0	0	0
1555A > G	3	3	0	0
1494C > T	0	1	0	0
IVS7-2A > G/-	0	7	0	0
IVS7-2A > G/IVS7-2A > G	2	7	0	0
2168A > G/-	1	3	1	0
2168A > G/IVS7-2A > G	1	1	0	0
235delC/35delG	4	0	0	0
235delC/299-300delAT	0	5	0	0
235delC/176-191del16	0	1	0	0
235delC/IVS7-2A > G	0	1	0	0
235delC/2168A > G	0	1	0	0
235delC/299-300delAT/ IVS7-2A > G	0	1	0	0
IVS7-2A > G/538C > T	0	1	0	0
299-300delAT/-	0	1	0	1
538C > T/-	0	1	0	0
187delG/187delG	3	0	0	0
1503G > A	1	0	0	0
Total	26	49	2	3

The frequency of *GJB2 *multination was 9.05% (18/199) in Uyghur patients, involving three mutations, namely, 235delC, 35delG, and 187delG. The allele frequencies were 3.52% (14/398), 2.26% (9/398) and 1.51% (6/398) in Uyghur patients, respectively. 235delC showed the highest frequency, whilst 187delG was identified for the first time in Uyghur NSD patients, and considered as an unreported pathological mutation of *GJB2*. The *GJB2 *mutation frequency in Han Chinese patients was 16.56% (25/151), also involving three mutations, namely, 235delC, 299-300delAT, and 176-191del16. The allele frequencies were 9.93% (30/302), 2.32% (7/302) and 0.33% (1/302) in Han Chinese patients, respectively. The mutation frequencies of *GJB2 *were 1.94% (2/103) and 4.28% (3/70) in Uyghur and Han Chinese control subjects (χ^2 ^= 4.495, *P *> 0. 05), respectively. The allele frequency of 235delC was significantly higher in Han Chinese patients than in Uyghur patients (χ^2 ^= 12.000, *P *< 0. 05), whereas that of 35delG was significantly higher in Uyghur patients than in Han Chinese patients (Fisher's exact probability test, *P *< 0. 05).

The frequencies of *SLC26A4 *mutation, including 2168A > G (H723R) and IVS7-2A > G, were 2.01% (4/199) in Uyghur patients and 14.57% (22/151) in Han Chinese patients, respectively. Of note, IVS7-2A > G mutant alleles accounted for 83.33% (25/30) of *SLC26A4 *mutation in Han Chinese patients. *SLC26A4 *mutation was less frequently found in Uyghur patients than in Han Chinese patients (χ^2 ^= 19.694, *P *< 0.05).

The frequencies of mtDNA 12S rRNA mutations, including 1555A > G and 1494C > T, were 2.01% (4/199) in Uyghur patients and 2.65% (4/151) in Han Chinese patients, respectively. The homozygous mutation of 1503 G > A was an unreported gene mutation. The mutation frequencies of 538C > T for *GJB3 *were 0.0% (0/199) in Uyghur patients and 1.32% (2/151) in Han Chinese patients. The mutation frequencies of mtDNA 12S rRNA and *GJB3 *were relatively low in both Uyghur and Han Chinese patients (Fisher's exact probability test, *P *> 0. 05).

### Mutation profiles of NSD patients with a definitive family history of deafness

Among Uyghur patients (n = 22) with a family history of deafness, the gene mutation in any of the nine loci studied was found in three (3/22, 13.64%) patients. In contrast, out of eleven Han Chinese patients with a family history of deafness, six patients (6/11, 54.55%) were found to carry the gene mutation in any of the nine loci. Most of these mutations were homozygous, homogenous, and double or compound heterozygous. For patients with a family history of deafness, the frequencies of gene mutations in the nine loci studied were significantly higher in Han Chinese patients than in Uyghur patients (Fisher's exact probability test, *P *< 0. 05).

## Discussion

In our study, the mutation frequencies of the nine loci for the four prevalent deafness-associated genes are 13.06% in Uyghur patients, 1.94% in Uyghur control subjects, 32.45% in Han Chinese patients, and 4.28% in Han Chinese control subjects, respectively. The mutation frequencies are significantly higher in NSD patients than in control subjects for either ethnicity, supporting the profound role of prevalent deafness-associated genetic testing in the etiological diagnosis of NSD patients. Moreover, the mutation frequency is significantly lower in Uyghur patients than in Han Chinese patients, requiring the further analysis of deafness-associated genes and mutation hotspots for the two ethnicities.

*GJB2 *gene encodes CX-26, a connexin associated with the formation of gap junction and osmolality. As Cx-26 is highly expressed on human cochlear hairy cells, *GJB2 *gene mutation is believed to be closely associated with NSD. *GJB2 *mutation prevails among deaf people across regions and races [17]. It is reported that *GJB2 *mutation accounts for over 50% of autosomal recessive inherited deafness cases [2,8]. Dai et al [18] reported that the frequency of *GJB2 *mutation was 4.0-30.4% in Chinese NSD patients. In our study, the mutation frequencies of *GJB2 *are 9.05% Uyghur patients and 16.56% for Han Chinese patients, in consistence with the report of Dai et al. In contrast to *SLC26A4*, mtDNA 12S rRNA and *GJB3, GJB2 *exhibits the highest mutation frequency in the two ethnicities, justifying that *GJB2 *remains the primary deafness-associated gene in both Uyghur and Han Chinese NSD patients. The mutant loci and allele frequencies differ significantly between the two ethnicities, whereas the overall mutation frequency of *GJB2 *mutation is comparable. Among the three mutations of *GJB2 *found in Uyghur patients, 235delC shows the highest allele frequency, and 35delG follows 235delC in allele frequency. 235delC is reported to be the major mutation among Eastern Asians [19-21], whereas 35delG is the hotspot mutation in Caucasians. Homozygous or heterozygous 35de1G mutation is also found in Western Asians, an extended family of Caucasian [22,23]. Both 235delC and 35delG are simultaneously identified as the predominant mutations in Uyghur NSD patients, which is probably attributed to the ethnic origin of Uyghur people. Located close to Central Asia, Kashi is an important stop of the ancient Silk Road. Uyghur people have mixed 30% of Caucasian kinship through the migration and cross-ethnic marriage, rendering themselves the Caucasian and Mongolian characters simultaneously [24]. Among the three mutations detected in Han Chinese NSD patients, 235delC has the highest allele frequency, consistent with the fact that 235delC is the major mutation in Eastern Asian population. In our study, 187delG is found in Uyghur NSD patients for the first time and considered as an unreported pathogenetic mutation of GJB2 (http://davinci.crg.es/deafness/, updated in January 2011). 187delG mutation results in the frameshift mutation of codon and the subsequent production of inactive connexin. Mutant proteins may cause the dysregulation of pH, decrease the permeability of gap junction, and consequently lead to hearing impairment. The allele frequency of 187delG is 1.51% (6/398), manifesting as homozygous mutations in the three Uyghur patients. Two of the three patients were borne by the couples in consanguineous marriages who have normal hearing otherwise. It suggests that all these parents are potential carriers. It is likely that 187delG is a common mutation among Uyghur NSD patients.

As a major sensorineural deafness-associated gene that is discovered following the identification of *GJB2, SLC26A4 *mutation is associated with the dilation of vestibular aqueduct in NSD [25] and accounts for 4-15% of inherited deafness cases [1,26]. In Western deaf population, L236P, T416P, E384G, and IVS8+1G > A are common mutant loci of *SLC26A4 *[21], whereas 2168A > G is the primary mutant locus in Japanese and Korean deaf patients [27,28]. IVS7-2 A > G is reported to be the major mutant locus of *SLC26A4 *in Han Chinese deaf population as well [15,29]. In our study, the two locus mutations of *SLC26A4*, namely, IVS7-2A > G and 2168A > G (H723R), are less frequently found in Uyghur NSD patients, whereas IVS7-2A > G is the primary mutation in Uyghur patients. Dai et al. [15] reported the complete absence of IVS7-2A > G mutation in 60 Uyghur patients. Li et al. [30] reported that IVS7-2A > G, a major mutant locus of *SLC26A4 *exhibited a relatively low frequency among Northwestern Chinese ethnicities, including Uyghur people. This finding shows a low carrier frequency of the two *SLC26A4 *locus mutations in Uyghur population. The mutation frequency of *SLC26A4 *is 14.57% (22/151) in Han Chinese NSD patients, whilst IVS7-2A > G mutant alleles account for 83.33% (25/30) among *SLC26A4 *mutation. This is consistent with the report that IVS7-2A > G is the primary mutation of *SLC26A4 *in Han Chinese NSD patients. The frequency of *SLC26A4 *mutation differs significantly between the two ethnicities. The low-frequency *SLC26A4 *mutation in Uyghur NSD patients is inconsistent with the fact that *SLC26A4 *is one of the major sensorineural deafness-associated genes. It can be inferred that Uyghur NDS patients differ from Mongolian ethnicity in the hotspot mutation of *SLC26A4*. Whether Uyghur patients share some mutation hotspots in *SLC26A4 *with Caucasian population remains yet to be investigated.

As a matrilineal mitochondrial gene, mtDNA 12S rRNA is associated with aminoglycoside-induced deafness [31,32]. 1555A > G and 1494C > T are the most important and common mutations. 1555A > G is more frequently detected, but shows a variable prevalence probably due to the ethnic variation in the susceptibility to aminoglycoside toxicity [33]. Both Spanish and Japanese severe progressive deaf populations show a relatively low-frequency 1555A > G mutation [34,35], in comparison with a frequency of 2.9-13.0% reported by Li et al. [11]. The mutation frequency of 1494C > T is even lower than that of 1555A > G. 1494C > T mutation is detected in only one Han Chinese patient in contrast to the 'zero' reported by Li et al. [11]. The mutation frequency of mtDNA 12S rRNA is statistically comparable between the two ethnicities. The unreported 1503G > A homogenous mutation that is found in one Uyghur NSD patient requires the further analysis to delineate whether it is a pathogenetic mutation.

*GJB3 *is a gene associated with NSD, and is initially cloned by Xia et al. [36]. *GJB3 *mutation is associated with the high-frequency hearing impairment in an autosomal dominant or recessive inheritance pattern [37]. *GJB3 *mutation is rarely detected in European and North American populations [38]. 538C > T mutation of *GJB3 *is detected in two of Han Chinese patients, but not in Uyghur patients or control subjects, showing a insignificant difference in the frequency of *GJB3 *mutation between the two ethnicities though.

The mutation frequencies of the nine loci in patients with a definitive family history of deafness are 13.64% for Uyghur and 54.55% for Han Chinese, respectively. The low mutation frequency in Uyghur NSD patients suggests that some mutations occurring at other loci or in other deafness-associated genes may be implicated in this ethnic group.

## Conclusion

Our study show that Uyghur NSD patients differ significantly from Han Chinese patients in deafness-associated gene mutation spectrum. Therefore, the further gene sequencing analysis and pedigree analysis are required to examine the mutation profile and determine the predominant mutations in Uyghur NSD patients.

## Competing interests

The authors declare that they have no competing interests.

## Authors' contributions

YC, HZ, and JS carried out the molecular genetic studies, participated in the collection of blood samples, and drafted the manuscript. MT, PK, and BH participated in the collection of blood samples. CH, HLL, and QS carried out the mutational analysis with PCR. DJ participated in the sequence alignment. YC and HZ participated in the design of the study, performed the statistical analysis, and drafted the manuscript. HZ and HLL reviewed and interpreted the results, drafted and revised the manuscript. All authors read and approved the final manuscript.
